# “Few” or “Many”? An Adaptation Level Theory Account for Flexibility in Quantifier Processing

**DOI:** 10.3389/fpsyg.2020.00382

**Published:** 2020-03-20

**Authors:** Stefan Heim, Natalja Peiseler, Natalia Bekemeier

**Affiliations:** ^1^Research Centre Jülich, Institute of Neuroscience and Medicine (INM-1), Jülich, Germany; ^2^Department of Psychiatry, Psychotherapy, and Psychosomatics, Medical Faculty, RWTH Aachen University, Aachen, Germany; ^3^JARA—Translational Brain Medicine, Aachen, Germany; ^4^Department of Linguistics, Heinrich Heine University Düsseldorf, Düsseldorf, Germany

**Keywords:** semantics, logic, quantities, degree, numerical cognition, linguistics

## Abstract

Quantifiers (e.g., “many,” “some,” “at least seven,” “more than half”) are words characterizing amounts or numerosities by reference to an internal threshold, or degree. For some quantifiers, this degree is not uniquely defined: It varies for external contexts (“many lions”/“many flies”) but may also be shifted within an individual (“many fries” for a hungry/full person). Previous studies showed that manipulation of the degree for one quantifier can impact that of other quantifiers. In this study, we tested whether such changes can occur by mere habituation, as formalized in the Adaptation Level Theory by Helson (1948) for sensory stimuli such as brightness or weight. To this end, participants read a quantifier statement and then judged whether a visual display with varying amounts (20–80%) of blue and yellow circles matched that statement. In Block 1, we identified which proportion of circles of a given color was judged by participants as “many” or “few.” In Block 2, we modified the presentation of stimuli such that (1) only the quantifier “many” was used and (2) only low proportions of circles of a given color were presented, thus changing the base rate at which proportions were encountered together with “many.” The hypothesis was that the internal degree of what is interpreted as “many” would be shifted downward and that this shift would also affect judgments of “few.” Block 3 was identical to Block 1, serving as a test for the expected effect on the degree/threshold for/across all proportions. The findings were as expected: The probability of accepting 40% as “many” was increased during Block 2, indicating adaptation. Likewise, the probability function for “few” was shifted in a parallel fashion around the proportion 40%. These findings complemented earlier studies demonstrating intra-individual flexibility in quantifier processing. They show that this flexibility can even be observed in the absence of explicitly stated verbal contexts or reinforcements, in line with the Adaptation Level Theory formulated originally for magnitudes, i.e., non-linguistic representations of quantities.

## Introduction

Humans can identify and distinguish physical stimulus intensities, magnitudes, and amounts of items. Moreover, they have mental representations thereof, and formal linguistic expressions for them, which are called “quantifiers.” Quantifiers are expressions of precise or vague quantities (e.g., *five, a couple*), groups or sets (e.g., *few*, *some*, *a lot, none*), or their relationships (e.g., *less than a quarter*, *the larger proportion, more than half*) (Barwise and Cooper, 1981). Whereas some quantifiers refer to an explicitly stated degree (=criterion), which can be absolute (e.g., 7 in *at most seven*) or relative (e.g., 25% in *a quarter*, independent on the base amount), others may have varying meanings, which are determined by context (e.g., Fernando and Kamp, 1996; Solt, 2011; Schöller and Franke, 2016; Schöller, 2017; see Spychalska et al., 2019, for slightly different interpretations of quantifiers depending on internal strategies). This context can be situational (e.g., *much water* in a jug of water vs. at high tide on the shore) and also internal (e.g., *much chocolate* when you study for an exam vs. when you want to lose weight before the summer; cf. Schöller and Franke, 2015). Yet, whatever the degree might be, there are consistencies in the preference functions over different contexts (Schöller and Franke, 2016). Moreover, humans tend to use quantifiers in a precise and informative way, maintaining an ordinal hierarchy roughly parallel to the mental number line (MNL) (e.g., Chater and Oaksford, 1999; Oaksford et al., 2002; Pezzelle et al., 2018; see also the multi-dimensional scaling approach by Routh, 1994): *None* is less than *few*, which is less than *some*, which is less than *many*—etc. up to *all* (see the probability heuristics model by Chater and Oaksford, 1999; see also Solt, 2011).

Within this rank order, the degree to which a particular quantifier pertains can be shifted when the internal context is modulated, e.g., by reinforcement learning (Heim et al., 2015, 2016). Reinforcement learning could make participants move their degree both up and down the mental number/probability line. Most importantly, this shift for one quantifier also affected its polar opposite: If the notion of *many*-ness was shifted downward (e.g., to already call 40% *many*), the notion of *few-*ness was affected in a complementary way. Likewise, if the degree for *few*-ness was shifted upward, this also affected the degree of its polar opposite *many* in a parallel fashion. The relevant brain area supporting this semantic re-evaluation of quantities was Broca’s region in the left inferior frontal cortex.

In the domain of sensory processing, such shifts in the evaluation of physical stimulus intensities have been described and formally explained for more than 70 years. The *Adaptation Level Theory* by Helson (1948) states that the central process is adaptation, i.e., habituation, to some physical intensity or magnitude. When we leave a dark room and enter open sunlight, we feel blinded by the brightness. Then, slowly, our sensory and neural systems adapt to the new intensity, which then becomes the default—the new *degree* in the terminology of quantifiers. When we re-enter the dark room, the formerly comfortable illumination in there will appear insufficiently little because of the new adaptation level from sunlight. Remaining in that room for a while will again lead to a shift back to the original adaptation level.

The objective of the present study was to bridge the gap between this theory in the sensory-perceptual domain and the linguistic-semantic domain, which relies on similar perception processes but requires additional cognitive evaluations to map one representation onto the other (e.g., Halberda et al., 2008; Pietroski et al., 2009; Szymanik and Zajenkowski, 2009, 2010; Lidz et al., 2011; Zajenkowski et al., 2011, 2014; Heim et al., 2012; Cheng et al., 2013; Odic et al., 2013; Zajenkowski and Szymanik, 2013). The crucial difference between the sensory-perceptual scenario and the linguistic-semantic experiment lies in the explicitness of the manipulation: Whereas sensory adaptation level shifts “happen” because of the exposure to varying degrees of stimulus intensities, the degree shift in the quantifier experiments (Heim et al., 2015, 2016) was (at least partly) introduced by explicit reinforcement learning. The participants gained or lost points (and thus money) if they did not judge the quantities according to the defaults imposed by the experimenter. Nevertheless, the transfer, or generalization, to the other quantifier was a process that occurred implicitly and without direct influence within the semantic network and along the hierarchically ordered axis of magnitude: When a magnitude formerly labeled *few* now qualifies as *many*, it cannot at the same time be *few* any longer (because in this case, two polar antonyms would be treated as synonyms, which, in turn, would create a contradiction and would reduce the relative informativeness of the quantifiers; cf. Chater and Oaksford, 1999; Oaksford et al., 2002; Pezzelle et al., 2018). These data are consistent with findings from a series of experiments on anchoring effects in quantifier processing (Sleeth-Keppler, 2013). When participants had received a numerical anchor before judging, e.g., the size of a famous building like the Eiffel Tower or the age of an (very young or very old) actor, the numerical size of the anchor systematically modulated the participants’ judgments.

This leads to the question of whether participants would change their quantifier degree in the same way as in the reinforcement experiments (Heim et al., 2015, 2016) if the experimental setting was a more naturalistic one as explained by Helson (1948), i.e., if the driving force was not brute force reinforcement but subtle habituation to a certain basic amount of objects. To be precise: In the reinforcement experiments, images of varying amounts of blue and yellow circles on a gray background were presented. Proportions of circles of a given color (e.g., yellow) ranged from 20 to 80%. Would participants shift their notion of *many*-ness (as applied to the case of circles of this color, and in this very same mode of presentation) if only a subset of these proportions was presented? Would, e.g., 40% of circles in yellow gradually fulfill the notion of *many*-ness if the maximum amount of yellow circles encountered in an experimental session did not exceed 50%?

The present study was an extension of the preliminary reinforcement studies, designed to answer this question. Using the same type of stimuli, pictures, and statements as in the previous reinforcement experiments, but combined a with a more naturalistic version of the paradigm, in which any habituation might happen eventually without explicit instruction or sanction, we sought to test how malleable quantifier semantics can be for one and the same empirical trial (a visual display of a particular proportion of colored circles and a sentence containing the quantifier “many” or “few”—the study does not extend to the investigation of implicature in the case of truth-value judgments for sets and subsets).

## Materials and Methods

The methods of this study were approved by the Ethics Committee of Heinrich Heine University Düsseldorf.

### Participants

Twenty-two healthy participants (22–36 years, mean 27.7 years; six men) took part in the study. All were native speakers of German. Twenty of the participants were right-handed, and the remaining two were left-handed.

### Stimuli and Procedure

We used a modification of the Truth Value Judgment Task (Oaksford et al., 2002; Heim et al., 2012) in which quantifier statements were presented (e.g., *Many of the circles are yellow*), followed by a visual display with blue and yellow circles of varying proportions (see examples in Heim et al., 2012, 2015, 2016). The participants’ task was to make a truth-value judgment, indicating by button press whether s/he thought that the statement was a true description of the visual display. As quantifiers, the words *many* and *few* were used; color words were *yellow* and *blue*. There were always 50 circles in one display, which were of varying diameters in order to prevent the total amount, or impression, of “yellow” or “blue” being strongly confounded with, and thus reliably indicative of, the actual number of circles of that color. Proportions ranging from 20 to 80% of the target color were used. The complement set of circles had the remaining color (e.g., 30% blue = 70% yellow).

Each trial began with the presentation of a fixation cross for 500 ms. Then a blank screen was presented for 100 ms. After that, the quantifier statement was presented to the participant auditorily over headphones. The statement’s duration was approximately 2000 ms.^1^ Next, the visual display with the 50 circles appeared on the screen until the decision was indicated by the button press. If no button was pressed, the picture disappeared after 3000 ms. Participants were required to indicate their truth-value judgment by pressing one of two response buttons as quickly as possible. Responses were recorded if they were made within a time window of 3000 ms. Otherwise, the trial was considered as not completed. Finally, the screen remained blank for 100 ms, resulting in a total trial duration of, at maximum, 6000 ms.

The experiment started with six training trials, during which participants could get familiar with the procedure of the experiment. Afterward, they had the chance to ask questions. The actual experiment consisted of three blocks. In Block 1, the individual preference of each individual to call a given percentage of circles of a given color *many* or *few* was determined (cf. Heim et al., 2015). In this block, the full range of proportions was used in order to get an unbiased estimate of the individual’s preferences. Block 1 consisted of a total of 112 trials, eight for each proportion for each of the two target colors.

In Block 2, the modulation of the adaptation level was introduced by limiting the proportions to the range of 20–50%. Moreover, only the quantifier *many* was contained in the initial statement. Participants continued to indicate their judgment as before. Critically, if their internal reference adapted to the new base rate of circles of the target color, lower proportions (in particular, 40%) should already qualify as *many* even though the initial preference for this judgment was low (Heim et al., 2015, 2016). Block 2 consisted of 168 trials, 21 for each proportion for each target color.

Finally, Block 3 was identical to Block 1, i.e., the full range of proportions was used again in 112 trials. Thus, Block 3 served as the test phase: If adaptation had taken place in Block 2, the judgments of the participants in Block 3 should differ significantly from those in Block 1 because of the shift of the internal degree.

### Data Analysis

The truth-value judgments were aggregated per participant, experimental block, quantifier, and proportion of circles of the target color. Next, the judgments for the critical proportion 40%^2^ were submitted to a 2 × 2 ANOVA with factors BLOCK (Block1 = baseline/Block3 = test) and QUANTIFIER (many/few). In addition, planned contrasts (*t*-tests, *p* < 0.05 one-tailed) were calculated. For the reaction times (RTs), an identical procedure was applied in order to establish whether the findings in the present study were comparable to those reported in previous works, in particular, the general observation of longer RTs for negative quantifiers. Thus, in the ANOVAs, a main effect for QUANTIFIER was expected for RTs but not for the truth-value judgments.

## Results

### Truth-Value Judgments

**FIGURE 1:**
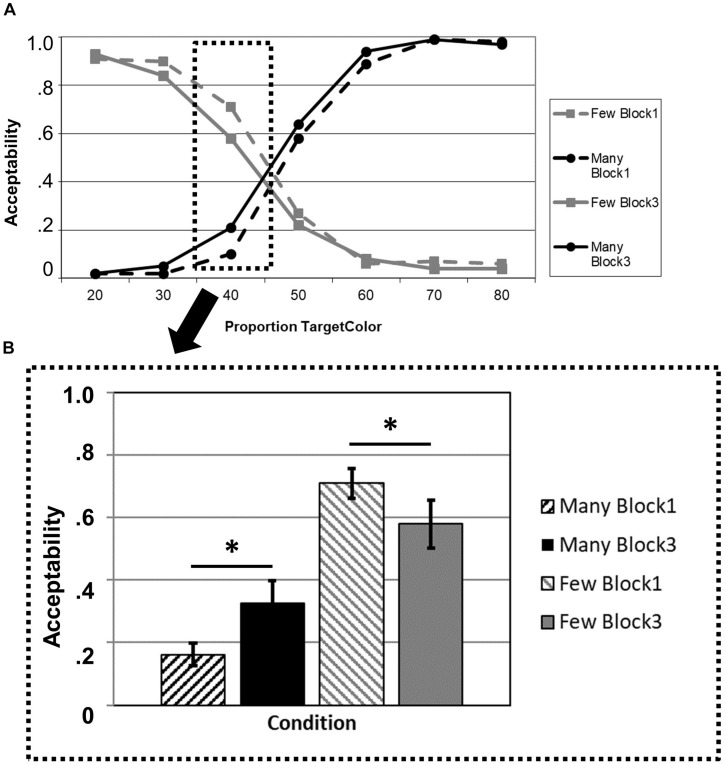
Acceptability ratings in the truth-value judgment task as a function of experimental block (Block 1: before adaptation; Block 3: after adaptation) and quantifier (*many, few*). **(A)** Overview of the full data set, including all proportions of the target color. **(B)** Values at the critical proportion 40%. The asterisk indicates significant differences at *p* < 0.05.

The overall pattern of truth-value judgments can be seen in Figure 1. The 2 × 2 ANOVA for the critical proportion 40% revealed a significant main effect of BLOCK (*F*_1_,_21_ = 27.862; *p* < 0.001; *η*^2^ = 0.570) and a significant interaction BLOCK × QUANTIFIER (*F*_1_,_21_ = 4.980; *p* = 0.037; *η*^2^ = 0.192). The main effect for QUANTIFIER was not significant (*F*_1_,_21_ = 0.167; *p* = 0.687; *η*^2^ = 0.008).

The *post hoc t*-tests yielded a significant effect for the quantifier *many* that had been solely presented in the adaptation phase (Block 2), demonstrating an increase in acceptability (*t*_21_ = 2.182; *p* = 0.021) in Block 3 compared to Block 1. Moreover, there was also an effect for the opposite quantifier *few* that had not been presented during the adaptation phase (*t*_21_ = 1.858; *p* = 0.039). That is, in Block 3, “few” was rated less acceptable for the proportion 40% than in Block 1. The full list of comparisons for the other proportions is provided in Table 1.

**TABLE 1:** Results of the pair-wise comparisons for truth-value judgments per proportion in Block 1 vs. Block 3 (all *p’s* one-tailed, uncorrected for multiple comparisons).

**Quantifier**	**Proportion**	***t*(21)**	***p***
**Many**	20	0	1
	30	–0.887	0.193
	40	–2.182	0.021
	50	–1.823	0.042
	60	–1.980	0.031
	70	0	1
	80	1.146	0.133

**Few**	20	–0.548	0.295
	30	1.502	0.074
	40	1.858	0.039
	50	1.521	0.072
	60	–0.636	0.266
	70	0.952	0.176
	80	0.979	0.170

### Reaction Times

**FIGURE 2:**
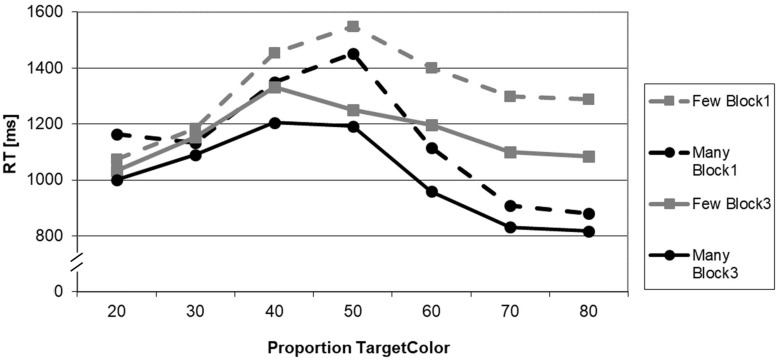
Reaction times in the truth-value judgment task as a function of proportion of the target color (in%), experimental block (Block 1: before adaptation; Block 3: after adaptation), and quantifier (*many, few*).

The pattern of RTs is presented in Figure 2. The 2 × 2 ANOVA for the critical proportion 40% revealed a significant main effect of BLOCK (*F*_1_,_21_ = 4.704; *p* = 0.042; *η*^2^ = 0.183) and a significant main effect for QUANTIFIER (*F*_1_,_21_ = 7.758; *p* = 0.011; *η*^2^ = 0.270). The interaction term BLOCK × QUANTIFIER was not significant (*F*_1_,_21_ = 0.108; *p* = 0.746; *η*^2^ = 0.005).

The *post hoc t*-tests yielded a significant effect for the quantifier *many*, i.e., that quantifier that had been solely presented and manipulated in the adaptation phase (*t*_21_ = 1.848; *p* = 0.040). Moreover, there was also an effect for the opposite quantifier *few* that had not been presented during the adaptation phase (*t*_21_ = 1.999; *p* = 0.030). In both instances, RTs decreased from Block 1 to Block 3. For a survey of the comparisons for all proportions, cf. Table 2.

**TABLE 2:** Results of the pair-wise comparisons for reaction times (RTs) per proportion in Block 1 vs. Block 3 (all *p’s* one-tailed, uncorrected for multiple comparisons).

**Quantifier**	**Proportion**	***t*(21)**	***p***
**Many**	20	2.519	0.010
	30	0.526	0.302
	40	1.848	0.039
	50	3.432	0.001
	60	2.333	0.015
	70	1.797	0.043
	80	1.562	0.067

**Few**	20	0.862	0.199
	30	0.396	0.348
	40	1.999	0.029
	50	3.764	0.001
	60	3.383	0.001
	70	3.244	0.002
	80	3.420	0.001

## Discussion

The present study investigated whether the variability of quantifier degrees, which can be observed in natural settings and which can be induced experimentally by reinforcement learning, would also occur eventually in an adaptation setting. The following pattern of results was found. (1) With respect to the acceptability ratings in the truth-value judgment task, exposition to a limited range of low proportions of the target color led to a shift in the acceptability of 40% as *many*. At the same time, there was also a parallel shift for *few*, which had not been present in the adaptation block. (2) In the domain of processing speed, RTs were always shorter in Block 3 than in Block 1, indicating a general habituation to the task setting. Additionally, RTs were always descriptively at maximum at the middle proportions, for which judgments could be considered to be more difficult. These findings will now be discussed in more detail.

This study bridges the gap between the sensory-perceptive and the linguistic-semantic domains of quantifier processing. So far, in the domain of semantics, it had been demonstrated that explicitly given verbal contexts can introduce mental expectations that, in turn, have an impact on how identical quantities or proportions of objects are evaluated, i.e., which quantifier expression is adequate and which is not (e.g., Fernando and Kamp, 1996; Solt, 2011; Schöller and Franke, 2015, 2016; Schöller, 2017). Moreover, it has been demonstrated that reinforcement learning can have an effect on the internal degree, which determines the adequateness of a quantifier, and that this learning had an impact not only on those quantifiers whose meaning was manipulated but also on the other quantifiers on the continuum (Heim et al., 2015, 2016). In the domain of sensory-perceptive processing, adaptation from one default level to a new default level had been observed and formally described (Helson, 1948). The question that had remained unanswered was whether such adaptation, which was connected to the first phase of quantifier processing and verification (Dehaene et al., 2003; Heim et al., 2012; Zajenkowski et al., 2014; see also Szymanik and Zajenkowski, 2010), would also occur in naturalistic learning environments without the presentation of explicit contexts, be they stated *a priori* to stimulus presentation or created by the reinforcement paradigm.

Given these preliminary studies, the results presented here are straightforward and as expected: Adaptation occurred in the acceptability ratings analogously to the explicit reinforcement setting, and it generalized from the one quantifier for which it had been induced to another quantifier—its polar opposite *few*. After the overall number of proportions was limited to the lower end of the spectrum (in Block 2), the probability of participants accepting 40% of something indeed to be many, not few, was statistically increased. Finally, the adaptation effect for “many” could also be observed at the neighboring proportions 50 and 60% (Table 1), whereas the generalization effect for “few” was smaller. This finding is perfectly consistent with the pattern of results in the direct reinforcement learning paradigms (Heim et al., 2015, 2016).

The RTs complement the picture. First, the main effect of QUANTIFIER nicely replicates the reports in the literature (Heim et al., 2012; Deschamps et al., 2015; Shikhare et al., 2015): The negative quantifier took, overall, longer to be processed. Second, the main effect of BLOCK revealed that the participants became more used to the task. Finally, the fact that RTs for the extreme proportions are shorter than for the intermediate proportions is known in the literature as the numerical distance effect (NDA; see, e.g., Moyer and Landauer, 1967): The more distant (and thus distinct) the numerosities, the lesser their overlap on the MNL and thus the higher the ease with which they can be distinguished. In consequence, the RT data demonstrate well-documented effects in the literature, thus serving as a quality check for the behavioral pattern as a whole and, in turn, for the interpretation of the truth-value judgment data.

To conclude, the data obtained here extend the notion of the flexibility of quantifier processing in cases where the degree is not fixed to one particular value. This is exactly the situation in which humans acquire quantifier meaning and its fine-tuning in the first place, i.e., during natural cognitive-linguistic development in early childhood (for a discussion, see Sullivan and Barner, 2011). The present findings also give rise to new research questions. For instance, would generalization also be observed for other quantifiers that are “neighbors” (cf. Oaksford et al., 2002; Pezzelle et al., 2018) on the continuum? Or is the seeming generalization merely driven by the fact that a quantifier other than *none* or *all* also has some link to its polar opposite because it also refers to the complement set (e.g., if some circles are yellow, some others are not; cf. Solt, 2011)? This question will have to be addressed in subsequent studies in which also the distinction between *many* with a cardinal meaning (i.e., reference to the degree zero) and *many* with a proportional meaning (in the sense of *many of*, i.e., reference to the particular set in question in a particular trial) might be tested (Schöller and Franke, 2016). Another interesting question is whether the adaptation effect observed here for colored geometrical shapes would also hold with real objects that have categorical semantics—and how long the adaptation effect may endure in these settings of varying abstractness. This information would be vital for potential clinical applications in cases in which a too-high or too-low internal degree may cause health issues: e.g., body weight in anorexia nervosa or obesity, or toxic amounts in cases of substance abuse. The present data can only be considered a first, tentative step into that direction. What they do show, however, is that adaptation effects as they occur in sensory perception may be induced in naturalistic ways without the need for explicit reinforcement.

Finally, it should be noted that the present study only tapped into one small and very particular aspect of the processing of magnitudes and numerosities, which is the verbal coding and its match to underlying representations. These representations themselves, access to these representations, and behavioral effects associated with accessing the representations are in the focus of a wealth of other studies (see, e.g., Weis et al., 2018; Nikolaev et al., 2020). Such studies make use of paradigms similar to the one used in the present study in that speeded two-choice reaction tasks are used. The tasks can require magnitude judgments or size compatibility effects and yield consistent and interestingly reliable behavioral patterns such as the Spatial Numerical Association of Response Codes (SNARC) effect. The focus of such studies is often on the compatibility of stimulus and response dimensions, e.g., whether the response speed of the left and right hands varies as a function of the physical or numerical magnitude of a stimulus on the screen (with small magnitudes favoring responses with the left hand = lower range of the MNL, and large magnitudes being associated with quicker responses of the right hand = upper range of the MNL). In the context of the present study, we did not analyze such compatibility effects, because the assignment of the responses to the response buttons was orthogonal to the physical size or numerosity of the stimulus: The subjects indicated the *truth value* of the combination of a quantified statement and a visual display. Thus, it was neither the amount of circles nor the polarity of the quantifier alone that determined the choice of the correct response button (left/right), but the combination. For that reason, we chose not to refer to literature on response compatibility in experimental settings similar to the one we used here (for a discussion of strategies of quantifier processing, the MNL, and response selection, see, e.g., Shikhare et al., 2015).

## Data Availability Statement

The datasets generated for this study are available on request to the corresponding author.

## Ethics Statement

The studies involving human participants were reviewed and approved by the Ethics Committee of Heinrich Heine University Düsseldorf. The patients/participants provided their written informed consent to participate in this study.

## Author Contributions

SH contributed to concept, study design, data analysis, discussion, and revision of manuscript. NP and NB contributed to concept, study design, data acquisition, discussion, and revision of manuscript.

## Conflict of Interest

The authors declare that the research was conducted in the absence of any commercial or financial relationships that could be construed as a potential conflict of interest.

## Footnotes

1 The color adjective was always at the end of the sentence. Thus, even though the processing of quantifier sentences is incremental (Urbach et al., 2015; Nieuwland, 2016), this procedure ensured that the mental representation/expectation had been created before the visual stimulus and, consequently, before the comparison and judgment process started.

2 At this proportion, acceptability rates start being meaningfully different from 0 in the baseline block (cf. Heim et al., 2012, 2015, 2016). One-sample *t*-tests in present study: 30%: *p* = 0.134, one-tailed; 40%: *p* = 0.004, one-tailed.
